# *Desulfovibrio* is not always associated with adverse health effects in the Guangdong Gut Microbiome Project

**DOI:** 10.7717/peerj.12033

**Published:** 2021-08-18

**Authors:** Yi-ran Chen, Qin-long Jing, Fang-lan Chen, Huimin Zheng, Li-dan Chen, Zhi-cong Yang

**Affiliations:** 1 Institute of Public Health, Guangzhou Medical University & Guangzhou Center for Disease Control and Prevention, Guangzhou, Guangdong, China; 2 Department of Intensive Care Unit, Shenzhen Second People’s Hospital, Shenzhen, Guangdong, China; 3 Department of Environmental Health, School of Public Health, Southern Medical University, Guangzhou, Guangdong, China; 4 Department of Laboratory Medicine, General Hospital of Southern Theatre Command of PLA, Guangzhou, Guangdong, China

**Keywords:** *Desulfovibrio*, Gut microbiota, Correlation, Host parameters, Microbial community

## Abstract

*Desulfovibrio* (DSV) is frequently found in the human intestine but limited knowledge is available regarding the relationship between DSV and host health. In this study, we analyzed large-scale cohort data from the Guangdong Gut Microbiome Project to study the ecology of DSV and the associations of DSV and host health parameters. Phylogenetic analysis showed that *Desulfovibrio piger* might be the most common and abundant DSV species in the GGMP. Predominant sub-OTUs of DSV were positively associated with bacterial community diversity. The relative abundance of DSV was positively correlated with beneficial genera, including *Oscillospira*, *Coprococcus*,*Ruminococcus*,*Akkermansia, Roseburia*,*Faecalibacterium*, and*Bacteroides*, and was negatively associated with harmful genera, such as *Clostridium*,*Escherichia*,*Klebsiella*, and *Ralstonia.* Moreover, the relative abundance of DSV was negatively correlated with body mass index, waist size, triglyceride levels, and uric acid levels. This suggests that DSV is associated with healthy hosts in some human populations.

## Introduction

*Desulfovibrio* (DSV) species are Gram negative species characterized by the ability to reduce sulphate to hydrogen sulfide in anaerobic respiration of organic matter (Gibson, Macfarlane & Cummings, 1988; Liamleam & Annachhatre, 2007). DSV species are widespread in natural environments (Gibson, Macfarlane & Cummings, 1988). In humans, DSV species can colonize the intestine, where high levels of organic nitrogen compounds support their growth (Gibson, Macfarlane & Cummings, 1988). Willis et al. (1997) found that human intestinal DSV could use alternative electron acceptors like sulfite, thiosulfate and nitrate, suggesting that sulfite, thiosulfate and nitrate in the diet could also influence the abundance of DSV.

Some DSV species are associated with disease. For example, Yachida et al. (2019) reported that *Desulfovibrio vietnamensis* and *D. longreachensis* increased in stage III/IV and stage 0 colorectal cancers, respectively. Bellocchi et al. (2018) reported that the gut microbiota of patients with systemic sclerosis was characterized by increased proinflammatory noxious genera, especially DSV. Crusell et al. (2018) reported that DSV abounded in women with gestational diabetes. Gobert et al. (2016) reported that DSV was higher in the gut microbiota of patients with constipated-predominant irritable bowel syndrome than in healthy people. Karlsson et al. (2012) reported that DSV was significantly lower in obese and overweight children than in normal-weight children. In contrast, Rowan et al. (2010) reported that the relative DSV load was associated with acute ulcerative colitis. Conversely, Hirano et al. (2018) reported fewer DSV at the inflammatory site of ulcerative colitis patients (*n* = 14) compared with the corresponding site of non-inflammatory bowel disease control (*n* = 14).

These contradictions may reflect sample size, inappropriate study subjects and the lack of subject health parameters. This suggests the need for large-scale cohort analysis.

In this study, we analyzed the gut microbiota data from the Guangdong Gut Microbiome Project (GGMP). This extensive gut microbiota dataset (He et al., 2018) contains 7009 individuals from 14 districts within Guangdong Province, China. We studied the prevalence and variation of DSV and the relationship between DSV and intestinal microbial community profile and evaluated the links between DSV and host parameters. Our results show that DSV was associated with healthy hosts in the GGMP dataset.

## Material and Methods

### Data acquisition and processing

Data acquisition and processing were applied as previously described for the GGMP (Chen et al., 2020). He et al. (2018) previously detailed and introduced GGMP. DNA was extracted from stool samples, and PCR amplification of the 16S rRNA V4 region and sequencing were conducted as described. Raw sequence data for the 16S rRNA gene are available from the European Nucleotide Archive at accession number PRJEB18535. These short reads were processed in the QIIME 2 framework using the Deblur denoising algorithm as previously described (Chen et al., 2020). A total of 6376 samples were remaining in the Deblur BIOM table for the follow-up analysis. Metadata for these samples can be found at https://www.nature.com/articles/s41591-018-0164-x. Deblur denoised sequences were assigned to bacterial features, which are almost equal to sub-OTUs (Wang et al., 2019). Taxonomic profiling of bacterial sub-OTUs was accomplished using the Greengenes reference database (version 13_8) as previously described (Chen et al., 2020).

### Phylogenetic analysis

Complete 16S rRNA gene sequences of six DSV type strains and two *Desulfobacter* type strains were obtained from the National Center for Biotechnology Information (NCBI). The V4 region of the 16S rRNA gene sequence was amplified with the barcoded V4 primers used for the GGMP (forward primer: 5′-GTGYCAGCMGCCGCGGTAA-3′, reverse primer: 5′-GGACTACNVGGGTWTCTAAT-3′) (He et al., 2018; Walters et al., 2016). We constructed a phylogenetic tree based on the V4 region gene sequences using MEGA 5, including eight predominant features of DSV (defined as a feature detected in more than 1% of all samples), six DSV-type strains (*D. piger* ATCC29098, *D. fairfieldensis* ATCC70045, *D. desulfurians* ATCC27774, *D. legallii* strain H1, *D. vulgaris* DSM644, and *D. intestinalis* strain KMS2), and two *Desulfobacter* type strains (*Desulfobacter postgatei* DSM2034 and *Desulfobacter vibrioformis* B54). GenBank accession numbers for each type strain are available in Table S1. The phylogenetic tree was constructed using MEGA (version 5.05) by neighbor-joining (ref). The test of phylogeny was performed using the bootstrap method, and the number of bootstrap replications was 1000. The gaps/missing data treatment was a complete deletion. Sequence alignment was calculated using the BLAST search at NCBI (https://blast.ncbi.nlm.nih.gov/Blast.cgi).

To further show the phylogenetic relationship of DSV features, we also constructed a tree based on the gene sequences of eight predominant features of DSV, and their best matches. This tree includes the V4 region gene sequences of eight predominant features of DSV, and the sequences of twenty strains. The parameters and model for this tree were same as above.

### Biostatistics analysis

To reveal the associations of DSV and predominant genera (defined as a genus with mean relative abundance above one percent) and the associations between predominant DSV features, we conducted a co-occurrence network analysis in R, as previously described (Chen et al., 2020). Spearman’s correlations were applied to each pair with FDR correction. Only significant correlations (FDR-adjusted *P* < 0.05) are shown.

Mann-Whitney U-tests were applied to compare DSV relative abundances in men and women, people with different BMI, normal waist and oversize waists, normal and elevated triglyceride (TG) levels, and normal and elevated uric acid (UA) levels. BMI and waist were classified on the basis of the Guidelines for Prevention and Control of Overweight and Obesity in Chinese adults. We classified TG levels on the basis of the Guidelines for the Prevention and Treatment of Dyslipidemia in Chinese adults. We classified UA levels according to the Chinese Guidelines for Diagnosis and Treatment of Hyperuricemia and Gout.

Kruskal-Wallis tests were performed to compute DSV relative abundance in seven Bristol stool types and at 14 geographical locations.

Correlations between Log_10_ relative abundance of DSV and *α*-diversity indices and correlations between the number of predominant DSV features and *α*-diversity indices were calculated by Spearman’s rank correlation test by SigmaPlot 13.0. Correlation between Log_10_ relative abundance of DSV and Log_10_ relative abundance of *Oscillospira* was calculated by Spearman’s rank correlation test and visualized using “ggpointdensity” (version 0.1.0) package. Correlations between DSV (at the genus and sub-OTU levels) and host metadata were calculated by Spearman rank correlation test, and FDR correction was conducted to adjust all *p*-values. A two-tailed *p*-value less than 0.05 was considered to have statistical significance for all analyses.

## Results

### Detection of *Desulfovibrio* in the GGMP samples

In total, we detected DSV in 3731 of 6376 gut microbiota. The mean relative abundance of the genus was 2‰. Seq14263 was the most prevalent. This sub-OTU was detected in 32% of all samples and accounted for 49% of the DSV-associated sequences (Table 1).

**Figure 1:**
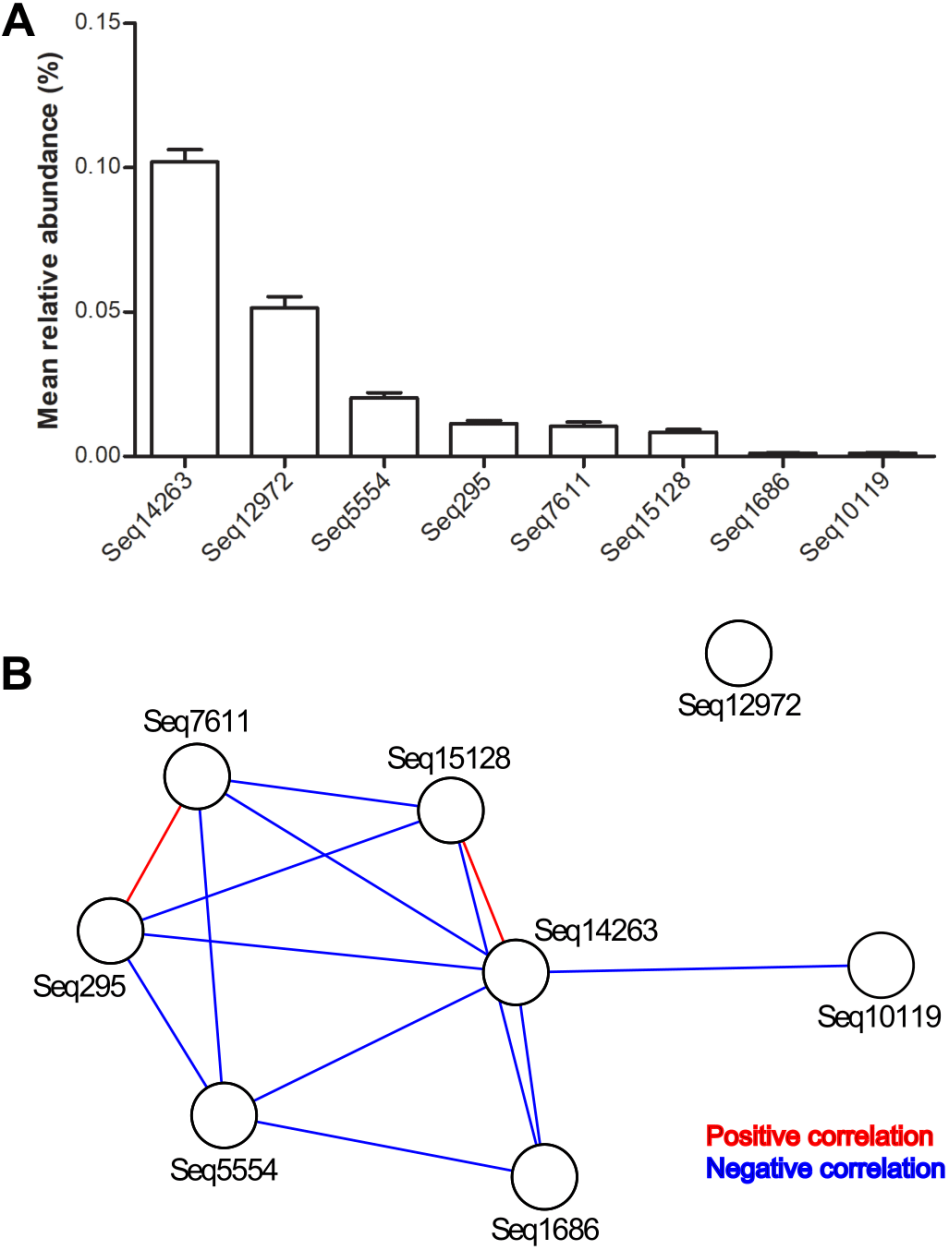
Relative abundances and internal associations of eight predominant DSV features. (A) Relative abundances of predominant DSV features across all samples, plotted by GraphPad Prism 5; (B) internal associations of the predominant DSV features. Red lines represent positive associations; blue lines represent negative associations.

The mean relative abundance of eight predominant features ranged from 0.01‰ to 1.02‰ (Table 1 and Fig. 1A). Among the total samples, 2831 samples carried only one predominant DSV feature, and 814 samples carried two or more predominant features. The maximum number of predominant DSV features detected in a sample was four, and only two samples carried four predominant DSV features. Co-occurrence analysis at the sub-OTU level showed that Seq14263 was positively associated with Seq15128. Seq7611 was positively associated with Seq295. Seq12972 was not associated with other predominant DSV sub-OTUs (Fig. 1B).

**Figure 2:**
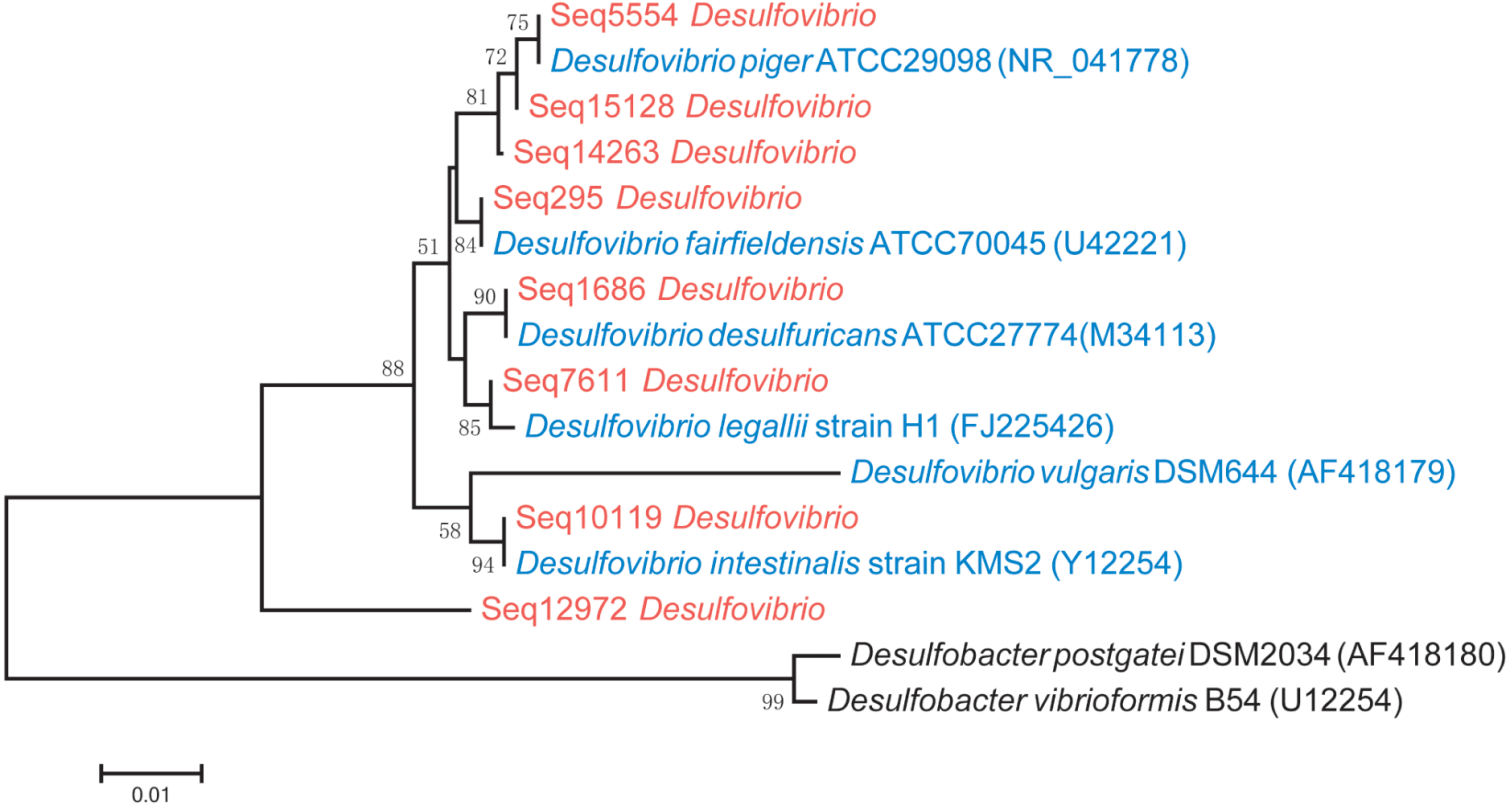
Phylogenetic tree of the eight predominant DSV features (marked in red), six typestrains of DSV (marked in blue) and two type strains of *Desulfobacter* (marked in black). The tree was constructed based on 16S V4 region sequences.

Phylogenetic analysis showed that *D. piger* might be the most common and abundant DSV species in the GGMP. The V4 regions of Seq295, Seq5554, Seq1686, and Seq10119 were 100% identical to that of *D. fairfieldensis* ATCC70045, *D. piger* ATCC29098, *D. desulfuricans* ATCC27774, and *D. intestinalis* strain KMS2, respectively. The V4 region of Seq12972 was not very similar (<93%, Table S3) to that of the six DSV type strains (Fig. 2). All the predominant sub-OTUs of DSV showed low similarity (<94%) to *D. vulgaris* DSM644 (Table S3), suggesting that it may be an uncommon strain in the GGMP.

A phylogenetic tree that includes the eight predominant features of DSV and twenty strains of DSV also showed that Seq14263, Seq15128 and Seq5554 clustered with *D. piger* strains with good (80%) bootstrap support (Fig. S1), suggesting that *D. piger* might be the most common and abundant DSV species. *D. fairfieldensis, D. desulfuricans, D. legallii*, *D. vulgaris*, and *D. intestinalis* were less prevalent in the GGMP.

### Microbial community profile link with *Desulfovibrio*

**Figure 3:**
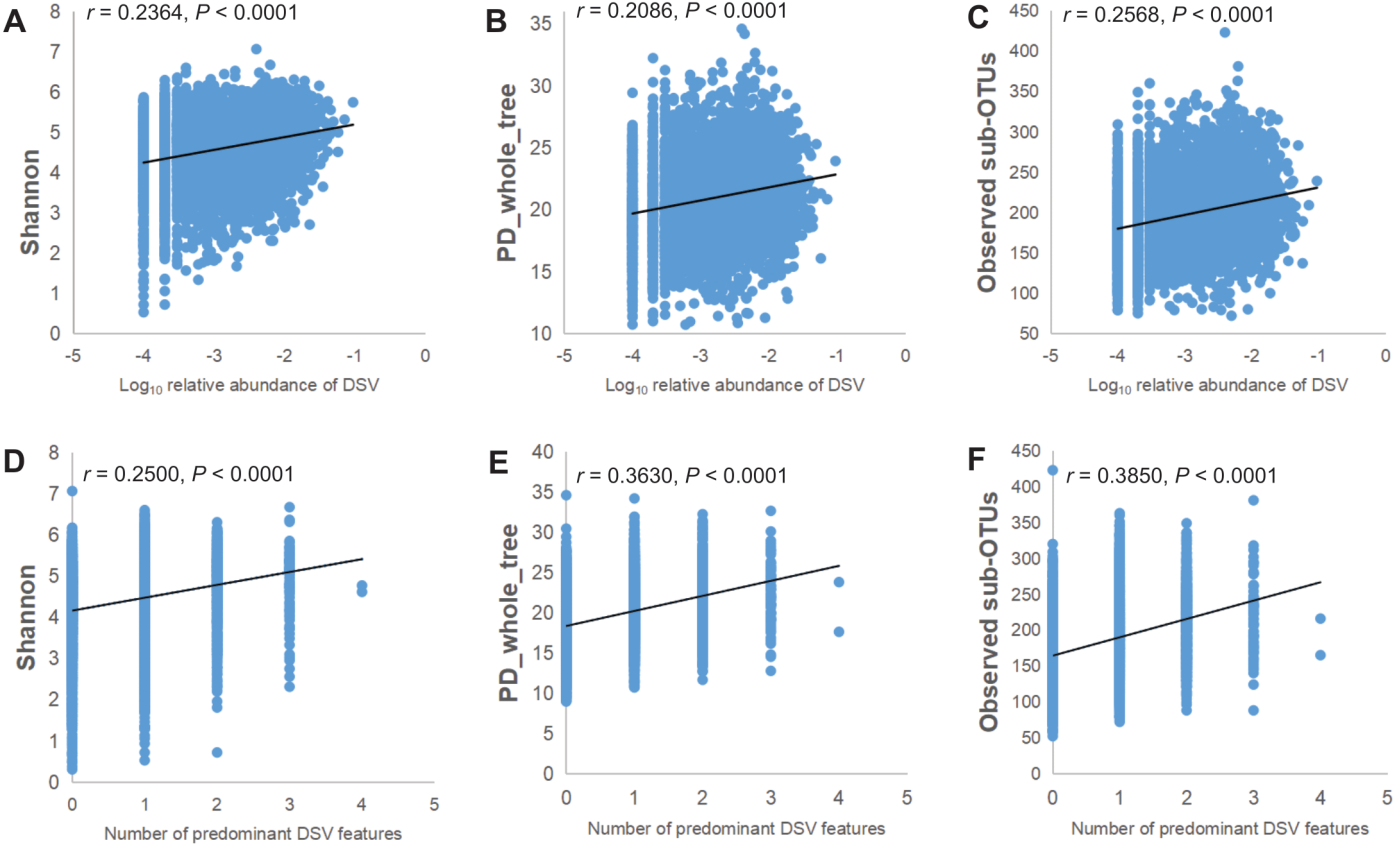
Microbial community *α*-diversity indices linked with DSV. (A–C) Spearman rank correlations between Log_10_ relative abundance of DSV and Shannon index,PD_whole_tree index, and Observed sub-OTUs. (D–E) Spearman rank correlations between the number of predominant DSV features and Shannon index, PD_whole_tree index, and Observed sub-OTUs.

The relative abundance of the genus of DSV was positively correlated with the microbiota *α*-diversity indices. As the Log_10_ relative abundance of the DSV genus increased, *α*-diversity also increased. The correlation between ascending *α*-diversity and DSV detection was positive when we examined *α*-diversity with Shannon, PD_whole_tree, and Observed sub-OTUs (Figs. 3A–3C). Spearman rank correlation analysis showed positive correlations between the number of predominant DSV features and Shannon, PD_whole_tree, and Observed sub-OTUs (Figs. 3D–3F).

**Table 1:** Prevalence, percentage, and mean relative abundance of predominant DSV features.

**Feature ID**	**Prevalence of predominant DSV features**	**Percentage of DSV-associated sequences**	**Mean relative abundance of predominant DSV features**
Seq14263	31.59%	48.50%	1.02 × 10^−3^
Seq12972	12.14%	24.45%	0.51 × 10^−3^
Seq5554	7.17%	9.64%	0.20 × 10^−3^
Seq295	7.64%	5.39%	0.11 × 10^−3^
Seq7611	3.98%	4.96%	0.10 × 10^−3^
Seq15128	5.46%	4.01%	0.08 × 10^−3^
Seq1686	1.71%	0.51%	0.01 × 10^−3^
Seq10119	1.41%	0.51%	0.01 × 10^−3^

**Figure 4:**
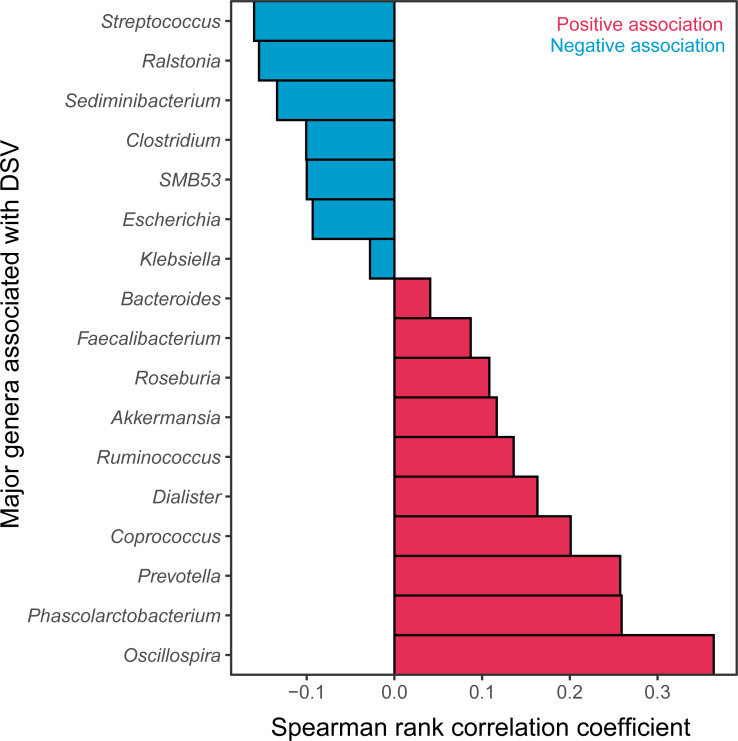
Major genera correlated with DSV. Red represents positive correlations; blue represents negative correlations.

The relative abundance of DSV was positively correlated with beneficial genera, and was negatively correlated with harmful genera. The relative abundance of DSV was positively associated with *Oscillospira*, *Phascolarctobacterium*, *Prevotella*, *Coprococcus*, *Dialister*, *Ruminococcus*, *Akkermansia*, *Roseburia*, *Faecalibacterium,* and *Bacteroides* and negatively associated with *Streptococcus*, *Ralstonia*, *Sediminibacterium*, *Clostridium*, *SMB53*, *Escherichia*, and *Klebsiella* (Fig. 4, Fig. S2). Correlation between DSV and *Oscillospira* was the strongest among all the correlations (Fig. 4, Table S4). Log_10_ relative abundance of DSV was positively correlated with Log_10_ relative abundance of *Oscillospira* in the GGMP in samples that DSV and *Oscillospira* can both be detected (Fig. S3A).

### Host parameters linked to the detection of *Desulfovibrio*

DSV relative abundance did not vary significantly with gender (*P* = 0.1559, Fig. S4A). The mean relative abundance of DSV was similar across samples with different Bristol stool types (*P* = 0.4879), ranging from 1.3‰ (type 7) to 2.4‰ (type 5) (Fig. S4B). There was a clear difference in the relative abundance of DSV among different geographical locations (*P* < 0.0001, Fig. S4C). DSV were most abundant in subjects from the Nanshan, Shenzhen (G440305), and least abundant in samples from subjects in the Wuchuan, Zhanjiang (G440883). The mean relative abundance of DSV ranged from 1.2‰ to 3.1‰ (Fig. S4C).

**Figure 5:**
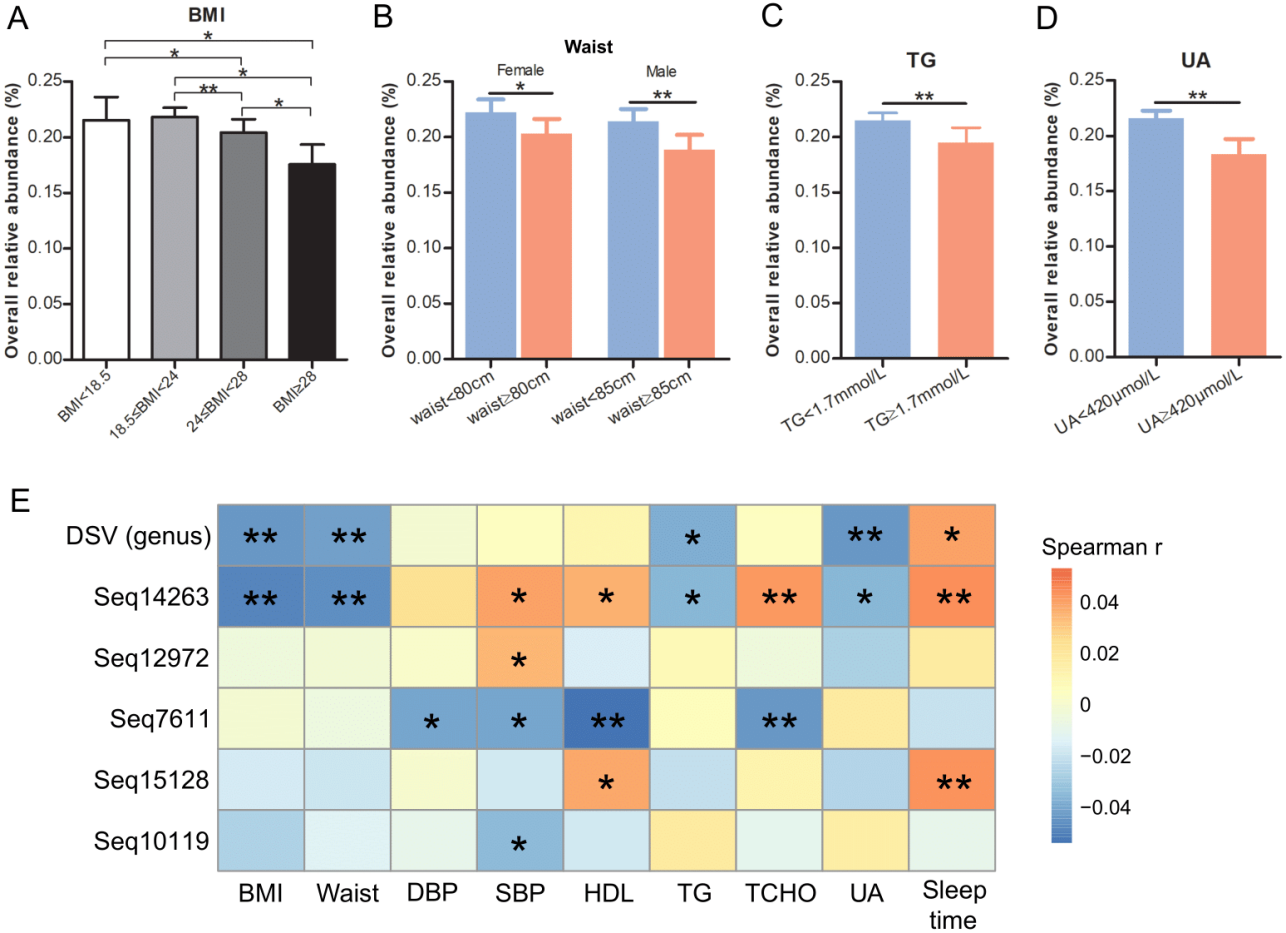
Relationships of DSV relative abundance and host metadata. Relative abundance of DSV in people with different BMIs (A), waist size (B), TG levels (C), and UA levels (D). (E) Heatmap of Spearman rank correlation coefficients gathering correlations between host parameters and DSV (at the genus and sub-OTU levels). An asterisk (*) indicates FDR adjusted *P* values smaller than 0.05; Two asterisks (**) indicate FDR-adjusted *P*-values smaller than 0.01; and three asterisks (***) indicate FDR-adjusted *P*-values smaller than 0.001.

DSV relative abundances in people with normal weight (18.5 ≤ BMI < 24) and low BMI (BMI < 18.5) were significantly more abundant than those in overweight (24 ≤ BMI < 28) and obese (BMI ≥ 28) (Fig. 5A). Women and men with normal waist sizes had higher levels of DSV than those with oversized waists (*P* = 0.0197 and *P* = 0.0015, respectively; Fig. 5B). The mean relative abundance of DSV was 2.2‰ *vs.* 2.0‰ in women and 2.1‰ *vs* 1.9‰ in men with normal and oversized waists, respectively (Fig. 5B). People with normal TG had higher levels of DSV than those with elevated TG (2.2‰ *vs.* 2.0‰, *P* = 0.0031, Fig. 5C). People with normal UA had higher levels of DSV than those with excessive UA (2.2‰*vs* 1.8‰, *P* = 0.0074, Fig. 5D).

At the genus level, DSV correlated negatively with host BMI, waist, TG, and UA. The mean relative abundances of DSV were 2.2‰ (low weight), 2.2‰ (normal weight), 2.0‰ (overweight), and 1.8‰ (obese). DSV genus and sleep time correlated positively (Fig. 5E). At the sub-OTU level, Seq14263 was negatively linked with BMI, waist size, TG, and UA and positively linked with systolic pressure (SBP), high-density lipoprotein (HDL), total cholesterol (TCHO), and sleep time; Seq12972 was positively linked with SBP; Seq7611 was negatively linked with diastolic pressure (DBP), SBP, HDL, and TCHO; Seq15128 was positively linked with HDL and sleep time; and Seq10119 was negatively linked with SBP (Fig. 5E, Table S5). Seq5554, Seq295, and Seq1686 were not correlated with these parameters. Low-density lipoprotein (LDL) was not correlated with DSV and these eight sub-OTUs (Table S5).

## Discussion

In this study, we revealed that DSV are generally associated with healthy hosts, which is in contrast to several previous studies. Petersen et al. reported the outgrowth of DSV was associated with obesity in mice. Increased DSV upregulated the expression of CD36, a receptor that mediates the binding to and uptake of long-chain fatty acids, thus promoting lipid absorption. These mouse model results correspond to reports that DSV are involved in diseases and adverse health effects (Vinke, El & Van Dijk, 2017; Kushkevych et al., 2019; Petersen et al., 2019; Rowan et al., 2010).

Our analysis of the GGMP suggest that DSV is not always associated with adverse health effects. First, DSV was negatively correlated with host BMI, waist, TG, and UA, which are all indications of obesity or metabolic disturbance (Osborne et al., 2020; He et al., 2018; Zeng et al., 2019; Tito et al., 2019). This is consistent with several previous studies. Karlsson et al. (2012) found that DSV were less abundant in obese/overweight children in Sweden. Andoh found that DSV were more abundant in lean people (range 31–58 years) than that in obese people (range 33–55 years) in Japan (Andoh et al., 2016). Second, DSV relative abundance was positively associated with microbial community diversity, which is conducive to microbiome stability and host health (Le Chatelier et al., 2013; Vieira-Silva et al., 2016). The number of predominant DSV features was also positively associated with microbial community diversity. Third, DSV was positively associated with *Oscillospira*, *Phascolarctobacterium*, *Prevotella*, *Coprococcus*, *Dialister*, *Ruminococcus*, *Akkermansia*, *Roseburia*, *Faecalibacterium*, and *Bacteroides* and negatively associated with *Streptococcus*, *Clostridium*, *Escherichia*, *Klebsiella*, and *Ralstonia. Oscillospira* is positively associated with lower BMI and lower levels of inflammatory diseases (Konikoff & Gophna, 2016). *Phascolarctobacterium* can generate short-chain fatty acids (Zhang et al., 2015) and is positively associated with positive mood in humans (Li et al., 2016). *Dialister* can be depleted in people with depression (Valles-Colomer et al., 2019). *Prevotella* is a beneficial genus because of its abundance in healthy human gut microbiota, although a few strains may have pathogenic potential (Precup & Vodnar, 2019). *Coprococcus*, *Ruminococcus*, *Akkermansia*, *Roseburia*, and *Faecalibacterium* produce short-chain fatty acid, which have health benefits (Duncan et al., 2002; Hiippala et al., 2018; Li et al., 2019; Plovier et al., 2017; Hou et al., 2020). *Bacteroides* species have health-promoting effects (Hiippala et al., 2018), while *Streptococcus*, *Clostridium*, *Escherichia*, and *Klebsiella* are generally considered harmful gut bacteria. Intestinal *Ralstonia* is more abundant in obese humans with T2DM and worsened glucose tolerance in diet-induced obese mice (Udayappan et al., 2017). Fourth, DSV relative abundance was weakly correlated with longer sleep time. Sleep depravation could disturb human microbiota and glycometabolism (Benedict et al., 2016).

An important factor in this dataset that could explain the positive associations between health effects and DSV is geographical location. He et al. (2018) showed that geography could affect human gut microbiota. Diet could also influence the host gut microbes. Gut microbes utilize components from food, and their metabolites may have beneficial or harmful effects on host physiology (Gentile & Weir, 2018). Ethnicity of the subjects in the GGMP study could be a factor. The ethnicity of subjects in this study was different from previous studies. Ethnicity relates to the gut microbiota (Gaulke & Sharpton, 2018). A study of 314 healthy volunteers from seven ethnic groups in China showed that gut microbiota composition at species level could be discriminated by the ethnicity (Zhang et al., 2015). Another study of 1673 volunteers in the United States showed that ethnicity could shape gut microbiota (Brooks et al., 2018).

In conclusion, based on an analysis of the GGMP dataset, we linked DSV with positive health parameters. This suggests DSV is beneficial for this specific population. We recommend more research to elucidate the relationship between DSV and host health in this population.

## Supplemental Information

10.7717/peerj.12033/supp-1 Supplemental Information 1 Phylogenetic tree of eight predominant DSV features and their best matches

10.7717/peerj.12033/supp-2 Supplemental Information 2 Co-occurrence network between DSV (marked in yellow) and the major genera Only the significant associations are shown. Red lines show positive associations, and blue lines show negative associations.

10.7717/peerj.12033/supp-3 Supplemental Information 3 (A) Spearman rank correlation between Log_10_ relative abundances of DSV and *Oscillospira* in the GGMP in samples that DSV and *Oscillospira* can both be detected. (**B**) Relative abundance of *Oscillospira* samples wit

10.7717/peerj.12033/supp-4 Supplemental Information 4 Relative abundances of DSV in males and females, people with different Bristol stool types, and people living in 14 geographical locations

10.7717/peerj.12033/supp-5 Supplemental Information 5 16S V4 region sequences of eight predominant DSV features, six type strains of DSV and two type strains of *Desulfobacter*

10.7717/peerj.12033/supp-6 Supplemental Information 6 16S sequences of six type strains of DSV and two type strains of *Desulfobacter*

10.7717/peerj.12033/supp-7 Supplemental Information 7 Sequence similarity of V4 region sequences

10.7717/peerj.12033/supp-8 Supplemental Information 8 The predominant genera (defined as the genera with mean relative abundance higher than 1%) associated with DSV tested by co-occurrence network analysis

10.7717/peerj.12033/supp-9 Supplemental Information 9 Spearman correlation coefficients summarizing correlations between host parameters and DSV at the genus and sub-OTU levels

10.7717/peerj.12033/supp-10 Supplemental Information 10 FDR adjusted *p* values of Spearman’s rank correlation between host parameters and DSV at the genus and sub-OTU levels

10.7717/peerj.12033/supp-11 Supplemental Information 11 FDR adjusted *p* values of Spearman’s rank correlation between blood pressure and DSV at the genus and sub-OTU levels for hypertensive individuals

10.7717/peerj.12033/supp-12 Supplemental Information 12 Sequences of eight predominant features of DSV, and their best matches

## Additional Information and Declarations

### Data Availability

The following information was supplied regarding data availability: The raw sequence data for the 16S rRNA gene are available at the European Nucleotide Archive: PRJEB18535. Data from the Guangdong Gut Microbiome Project were published in Table S13: He, Y., Wu, W., Zheng, HM. et al. Regional variation limits applications of healthy gut microbiome reference ranges and disease models. Nat Med 24, 1532–1535 (2018). https://doi.org/10.1038/s41591-018-0164-x. Reprinted by permission from [Springer Nature BV]: [Nature Research] [Nature medicine] [He, Y., Wu, W., Zheng, HM. et al. Regional variation limits applications of healthy gut microbiome reference ranges and disease models. Nat Med 24, 1532–1535 (2018).
