# Supplementary figures and images for: Desulfovibrio is not always associated with adverse health effects in the Guangdong Gut Microbiome Project
Source: PeerJ. 2021 Aug 18;9:e12033. doi: 10.7717/peerj.12033 (PMC8380029; doi:10.7717/peerj.12033)

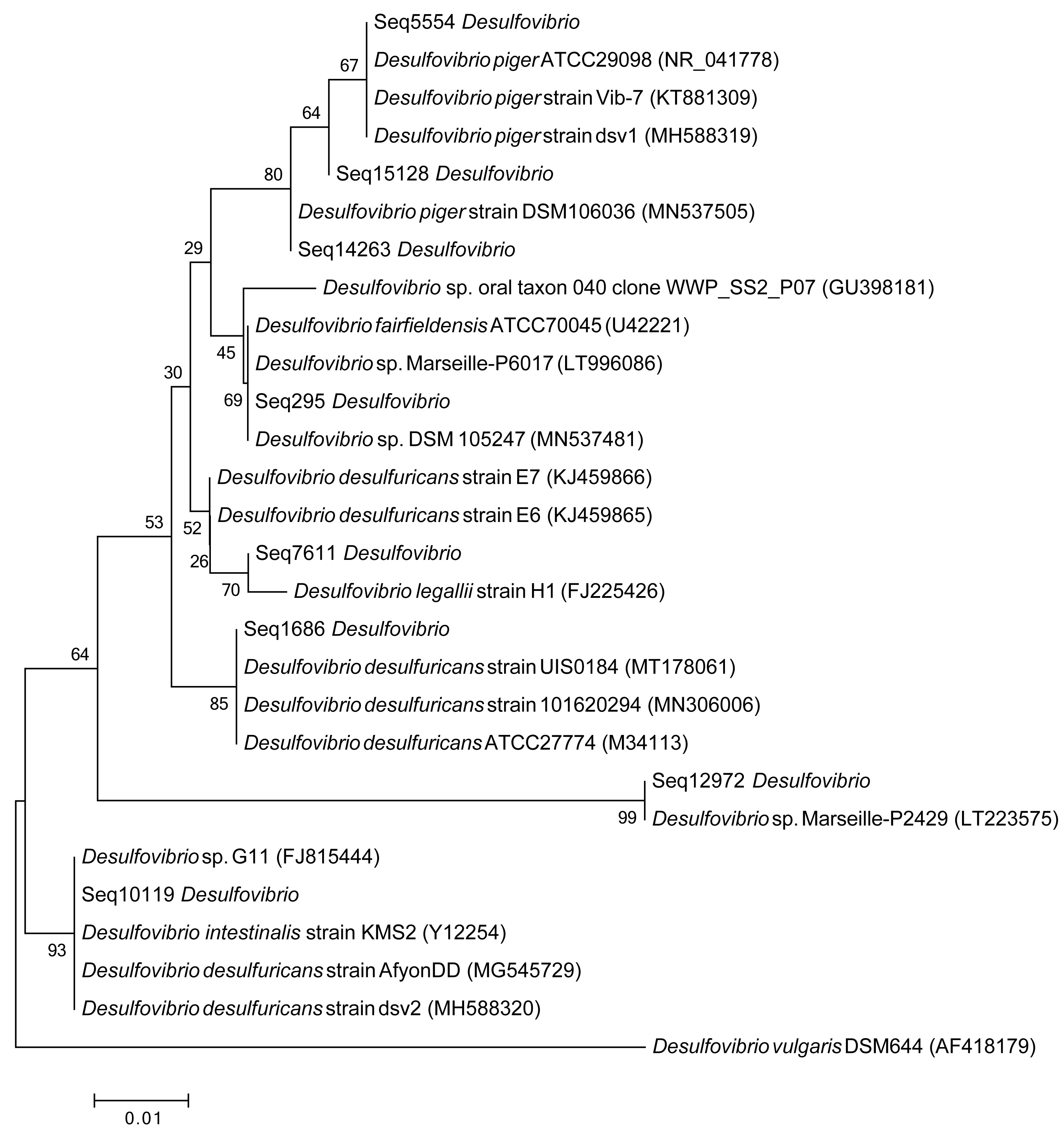

Supplement: Supplemental Information 1 [file peerj-09-12033-s001.jpg]

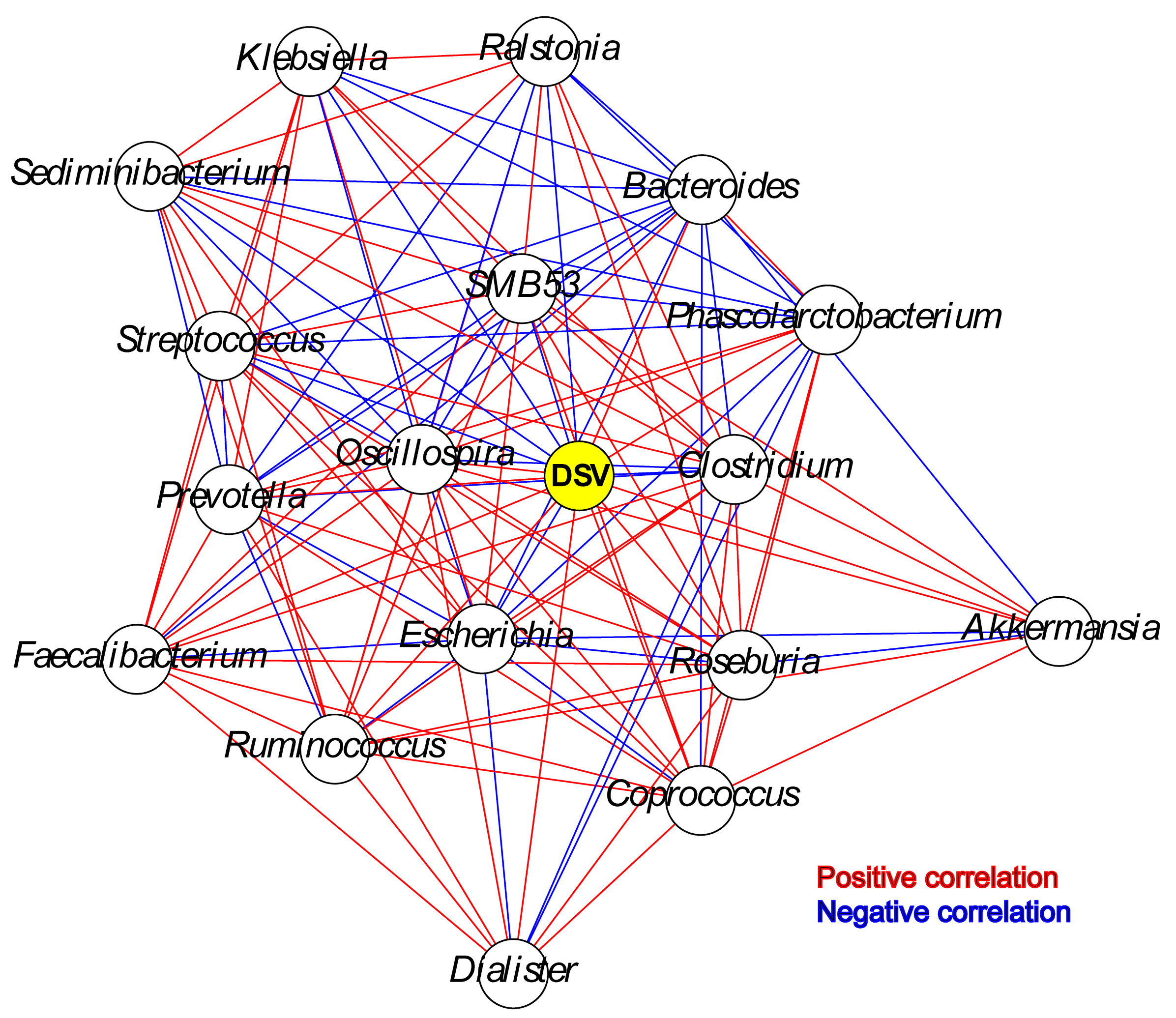

Supplement: Supplemental Information 2 — Only the significant associations are shown. Red lines show positive associations, and blue lines show negative associations. [file peerj-09-12033-s002.jpg]

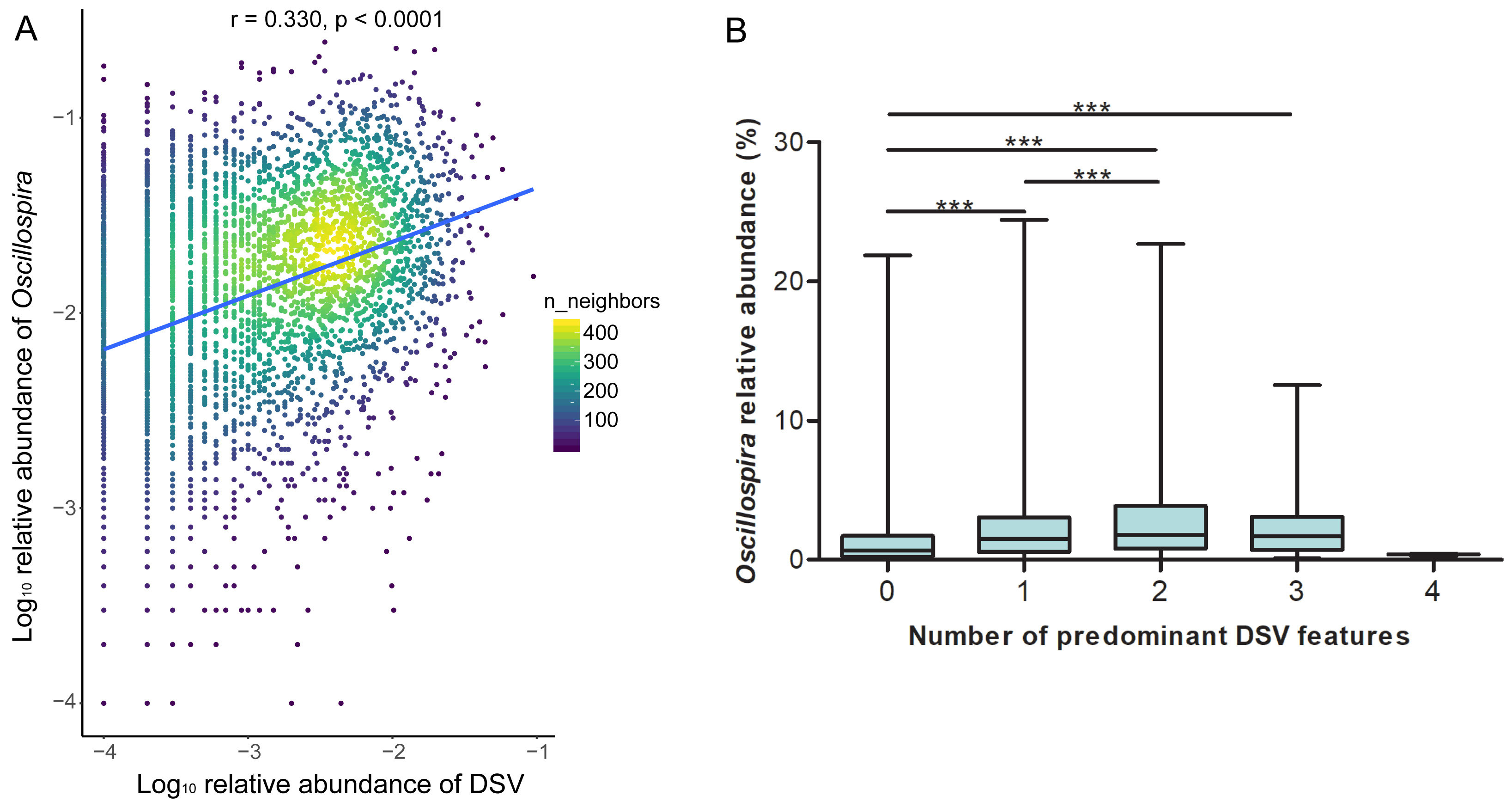

Supplement: Supplemental Information 3 [file peerj-09-12033-s003.jpg]

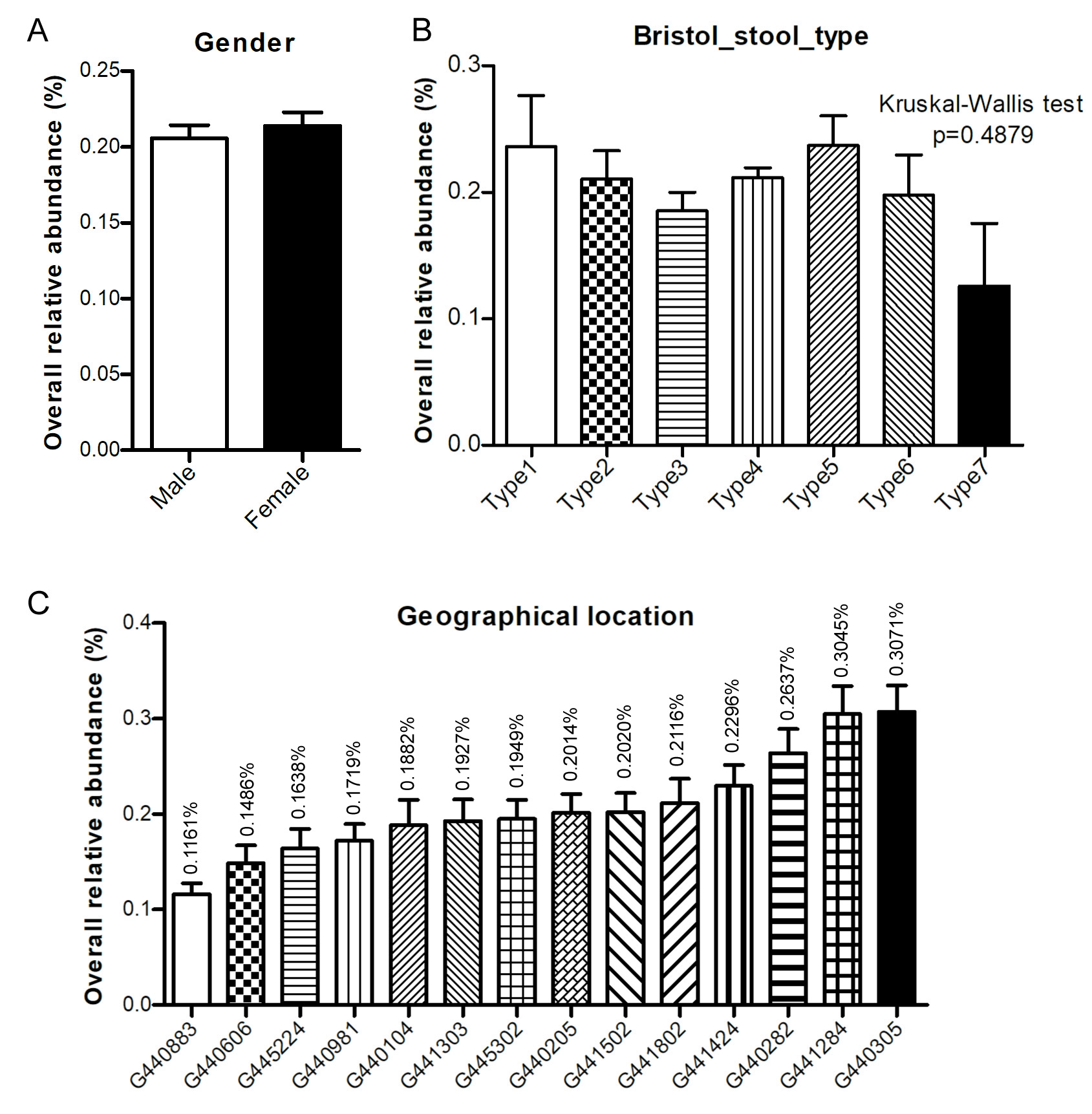

Supplement: Supplemental Information 4 [file peerj-09-12033-s004.jpg]
